# Supplementary material for: Novel combination of CRISPR-based gene drives eliminates resistance and localises spread
Source: Sci Rep. 2021 Mar 4;11:3719. doi: 10.1038/s41598-021-83239-4 (PMC7933345; doi:10.1038/s41598-021-83239-4)
Supplement: Supplementary file 1 — Supplementary Information [file 41598_2021_83239_MOESM1_ESM.pdf]

Novel combination of CRISPR-based gene drives eliminates resistance and localises spread

Nicky R. Faber[1, 2, *], **Gus R. McFarlane**[3], **R. Chris Gaynor**[4], **Ivan Pocrnic**[1], **C. Bruce A. Whitelaw**[3], **and Gregor Gorjanc**[1]

[1]Highlander Lab, The Roslin Institute and Royal (Dick) School of Veterinary Studies, The University of Edinburgh, Easter Bush Campus, Midlothian EH25 9RG, United Kingdom
[2]Laboratory of Genetics, Wageningen University & Research, Droevendaalsesteeg 4, 6708PB Wageningen, The Netherlands
[3]Whitelaw Group, The Roslin Institute and Royal (Dick) School of Veterinary Studies, The University of Edinburgh, Easter Bush Campus, Midlothian EH25 9RG, United Kingdom
[4]AlphaGenes Group, The Roslin Institute and Royal (Dick) School of Veterinary Studies, The University of Edinburgh, Easter Bush Campus, Midlothian EH25 9RG, United Kingdom
[*]nfaber@outlook.com

ABSTRACT

Invasive species are among the major driving forces behind biodiversity loss. Gene drive technology may offer a humane, efficient and cost-effective method of control. For safe and effective deployment it is vital that a gene drive is both self-limiting and can overcome evolutionary resistance. We present HD-ClvR, a novel combination of CRISPR-based gene drives that eliminates resistance and localises spread. As a case study, we model HD-ClvR in the grey squirrel (*Sciurus carolinensis*), which is an invasive pest in the UK and responsible for both biodiversity and economic losses. HD-ClvR combats resistance allele formation by combining a homing gene drive with a cleave-and-rescue gene drive. The inclusion of a self-limiting daisyfield gene drive allows for controllable localisation based on animal supplementation. We use both randomly mating and spatial models to simulate this strategy. Our findings show that HD-ClvR can effectively control a targeted grey squirrel population, with little risk to other populations. HD-ClvR offers an efficient, self-limiting and controllable gene drive for managing invasive pests.

Supplementary figures

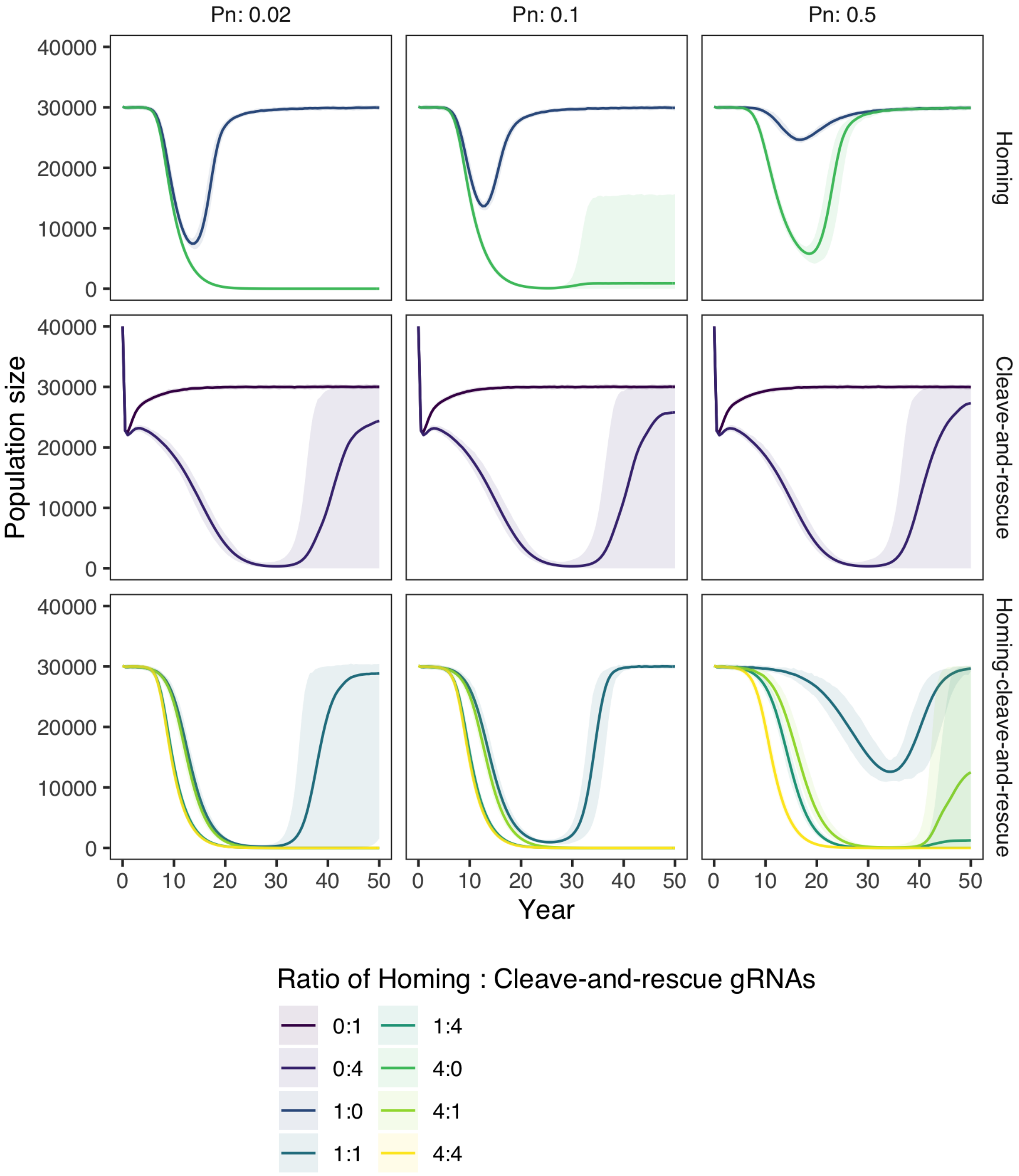

Figure S1. The same as Figure 3, but run in a big population with a carrying capacity of 30,000 instead of 3,000. Population size over time after the introduction of gene drive squirrels with either a standard homing, a standard cleave-and-rescue, or a homing-cleave-and-rescue gene drive to a population with carrying capacity 30,000. All simulations are based on a single release of 100 squirrels is done, other than the standard cleave-and-rescue gene drive, which requires a release of 10,000 squirrels. Lines represent the average population size over 100 model replications, while opaque ribbons represent the 95% quantiles. The model was run with 3 different rates of NHEJ repair during homing (*Pn*) and with different numbers of gRNAs for the homing and the cleave-and-rescue components of the gene drive.

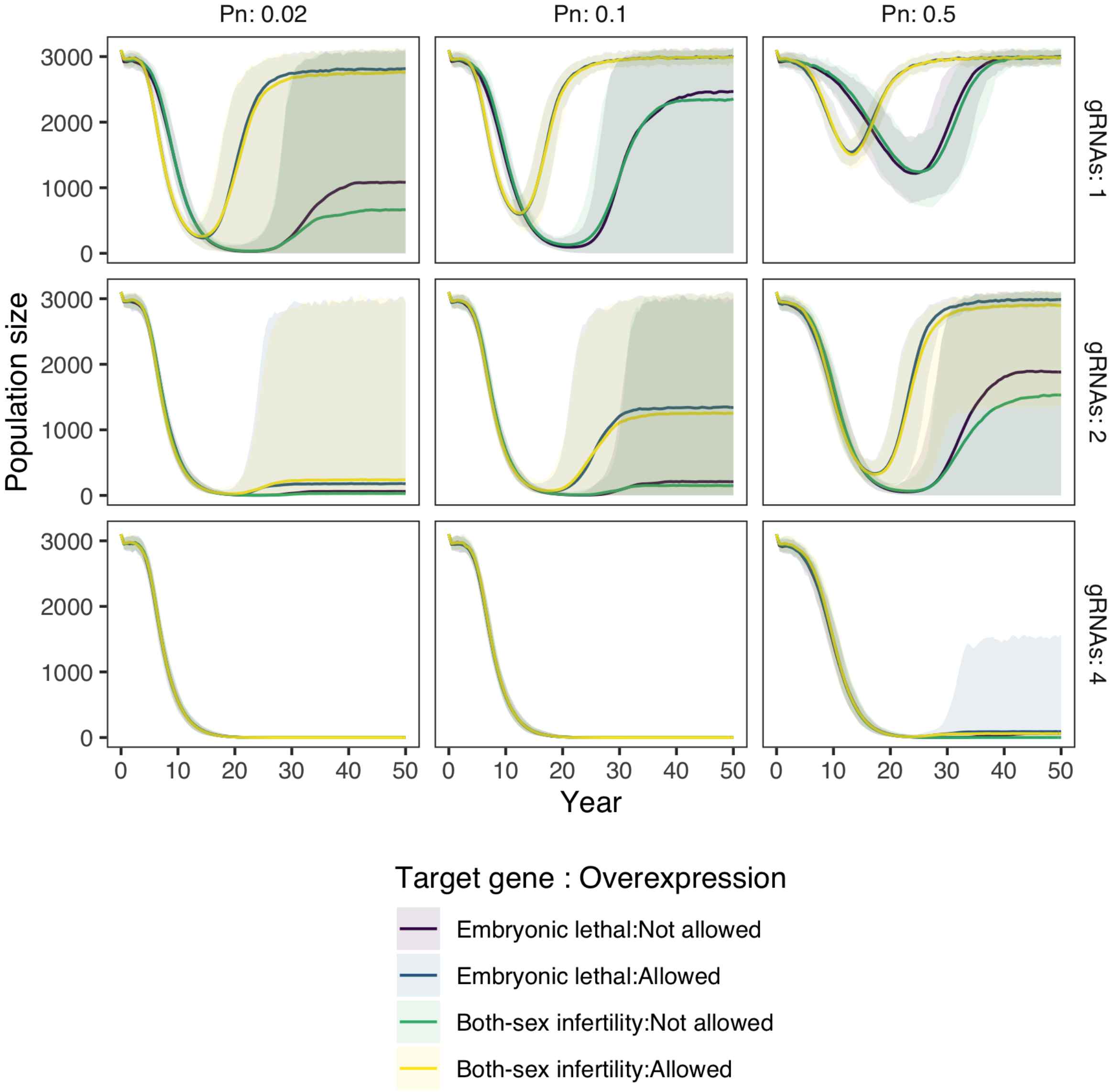

Figure S2. An exploration of which type of gene is best targetted by the cleave-and-rescue part of the gene drive: both-sex infertility or developmental non-viability, and overexpression biologically tolerable or not. Parameters are kept the same as in Figure 3, except that we used 1 gRNA for the homing part of the gene drive, and either 1, 2 or 4 gRNAs for the cleave-and-rescue part. Population size over time after the introduction of 100 gene drive squirrels with a homing-cleave-and-rescue gene drive to a population with carrying capacity 3,000. Lines represent the average population size over 100 model replications, while opaque ribbons represent the 95% quantiles. The model was run with 3 different rates of NHEJ repair during homing (*Pn*).

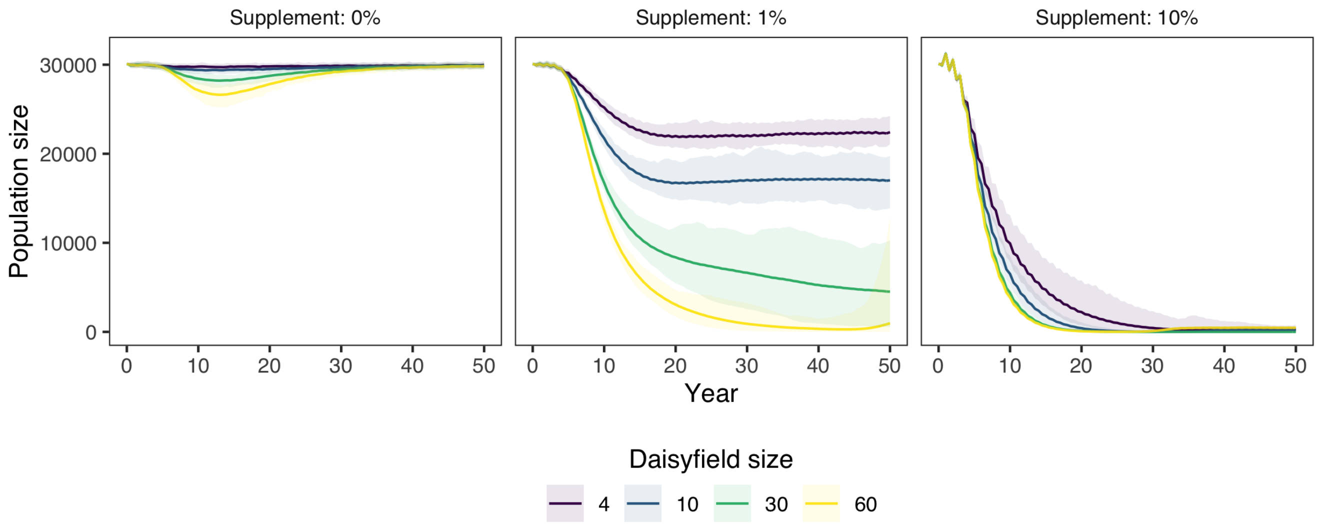

Figure S3. The same as Figure 4, but run in a big population with a carrying capacity of 30,000. Population size over time after the introduction of 100 squirrels with a HD-ClvR gene drive. The model was run with an NHEJ rate (*Pn*) of 0.02, 1 homing gRNA, and 4 cleave-and-rescue gRNAs. Gene drive squirrel supplementation was done yearly, the amount being a percentage (0, 1, or 10%) of the total population size at that moment. Lines represent the average population size over 100 model replications, while opaque ribbons represent the 95% quantiles.

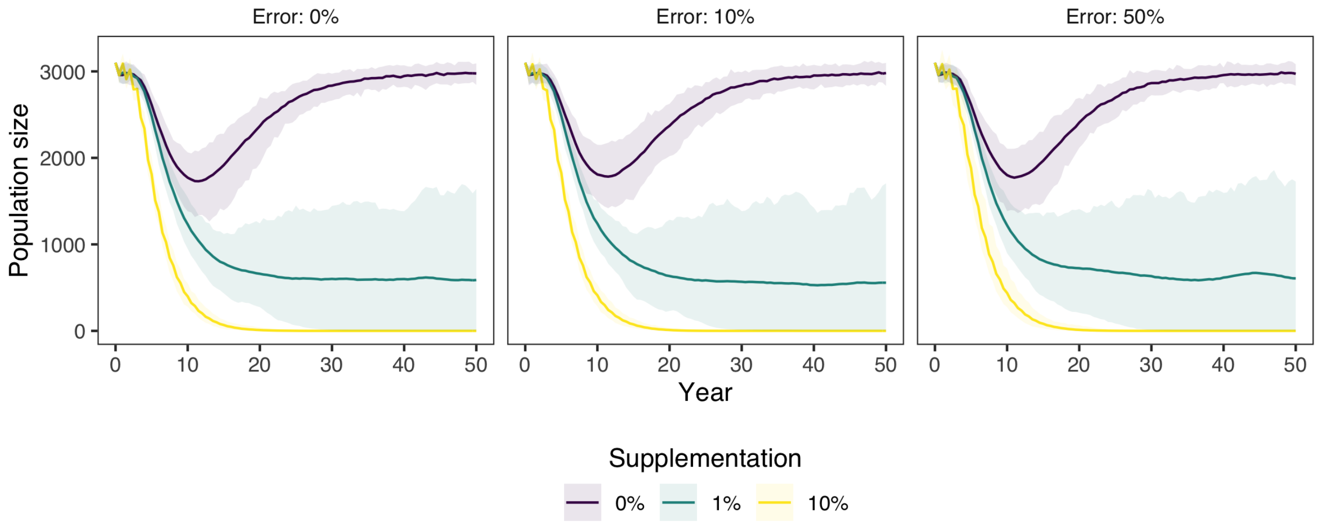

Figure S4. The same as Figure 4, but instead of an accurate estimate of the population size for supplementation, a certain level of error is introduced. The error is defined on a yearly basis as a normal distribution with the true population size as mean and a certain percentage of the true population size as standard deviation. Population size over time after the introduction of 100 squirrels with a HD-ClvR gene drive to a population of carrying capacity 3,000. The model was run with an NHEJ rate (*Pn*) of 0.02, 1 homing gRNA, and 4 cleave-and-rescue gRNAs. Gene drive squirrel supplementation was done yearly, the amount being a percentage (0, 1, or 10%) of the total population size at that moment, plus the abovementioned error. Lines represent the average population size over 100 model replications, while opaque ribbons represent the 95% quantiles.

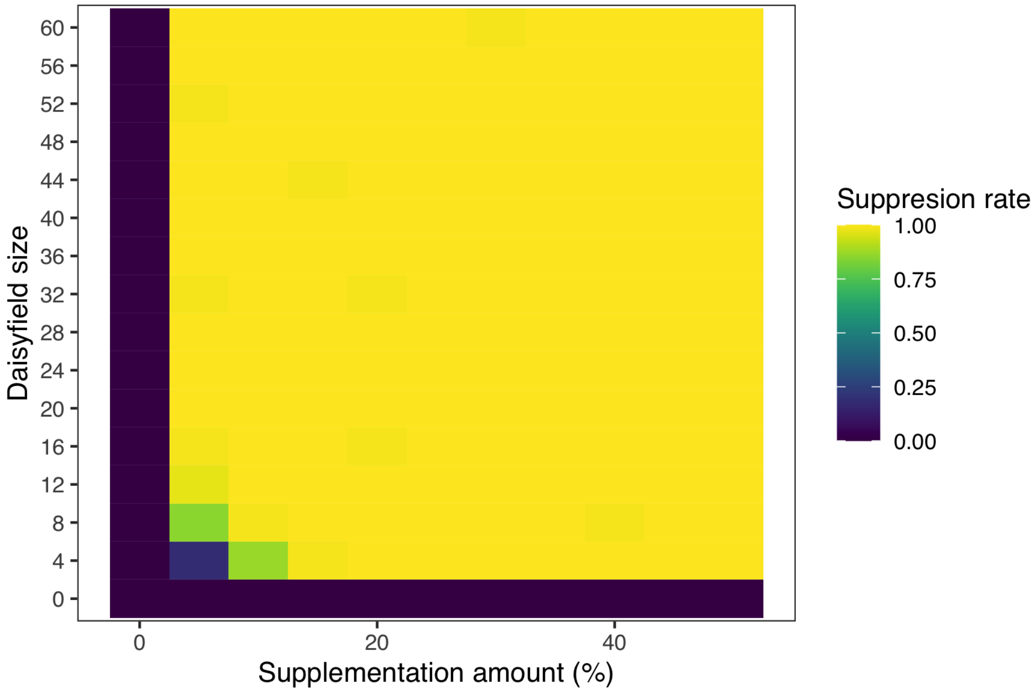

Figure S5. The same as Figure 4, but ran with a range of supplementation amounts and daisyfield sizes. Suppression rate is defined as the proportion of populations (out of the 100 repetitions of the model) that were completely suppressed after 50 years. Suppression rate after the introduction of 100 squirrels with a HD-ClvR gene drive to a population of carrying capacity 3,000. The model was run with an NHEJ rate (*Pn*) of 0.02, 1 homing gRNA, and 4 cleave-and-rescue gRNAs.

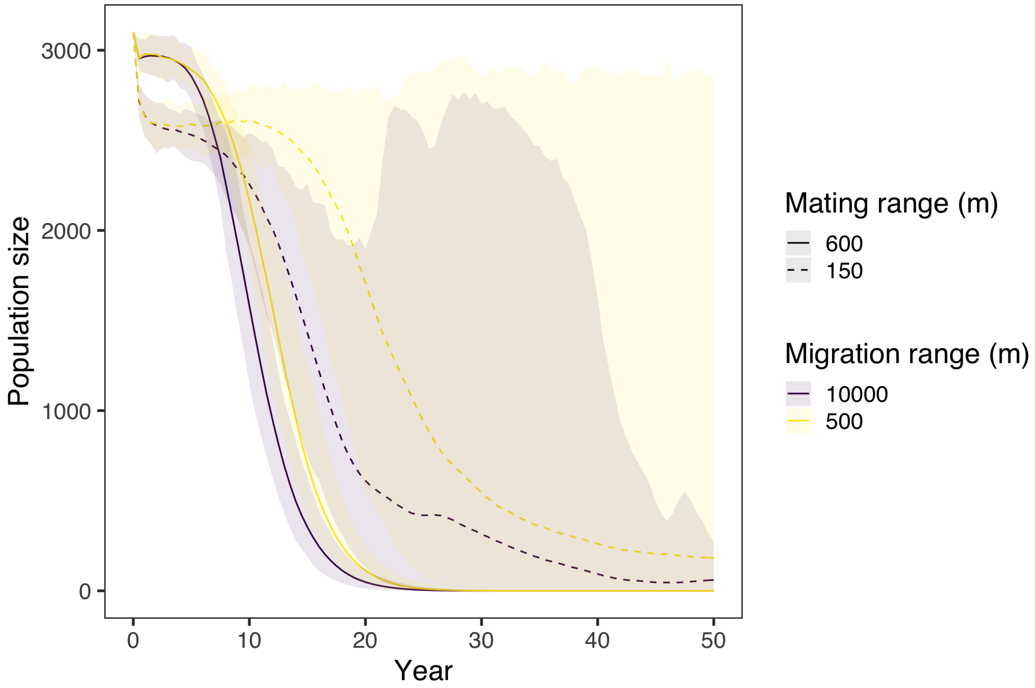

Figure S6. A sensitivity analysis of two crucial parameters in our spatial model (Figure 5): mating range and migration range. We model population size over time after the introduction of 100 squirrels with a homing-cleave-and-rescue gene drive with 1 homing gRNA and 4 cleave-and-rescue gRNAs. An NHEJ rate (*Pn*) of 0.02 was used. In the spatial model, gene drive squirrels were placed in the middle of the area. Lines represent the average population size over 100 model replications, while opaque ribbons represent the 95% quantiles.

Figure S7. Full animations of the spatial dynamics of HD-ClvR using the five placement schemes shown in Figure 5 (see https://git.ecdf.ed.ac.uk/HighlanderLab_public/nfaber_squirrel_gd/tree/master/Fig6_GIFs). We model the spatial dynamics of a population over time after the introduction of 100 squirrels with a HD-ClvR gene drive with 1 homing gRNA and 4 cleave-and-rescue gRNAs to a population of carrying capacity 3,000. We modelled an NHEJ rate (*Pn*) of 0.02, a daisyfield of size 30 and a supplementation amount of 10%.
